# Identification of cardiac hemo-vascular precursors and their requirement of sphingosine-1-phosphate receptor 1 for heart development

**DOI:** 10.1038/srep45205

**Published:** 2017-03-24

**Authors:** Yan Hu, Brian C. Belyea, Minghong Li, Joachim R. Göthert, R. Ariel Gomez, Maria Luisa S. Sequeira-Lopez

**Affiliations:** 1 Department of Pediatrics and Department of Biology, University of Virginia, Charlottesville, Virginia, USA; 2 Department of Hematology, West German Cancer Center, University Hospital Essen, Essen, Germany

## Abstract

The cardiac endothelium plays a crucial role in the development of a functional heart. However, the precise identification of the endocardial precursors and the mechanisms they require for their role in heart morphogenesis are not well understood. Using *in vivo* and *in vitro* cell fate tracing concomitant with specific cell ablation and embryonic heart transplantation studies, we identified a unique set of precursors which possess hemogenic functions and express the *stem cell leukemia (SCL*) gene driven by its 5′ enhancer. These hemo-vascular precursors give rise to the endocardium, atrioventricular cushions and coronary vascular endothelium. Furthermore, deletion of the *sphingosine-1-phosphate receptor 1 (S1P1*) in these precursors leads to ventricular non-compaction cardiomyopathy, a poorly understood condition leading to heart failure and early mortality. Thus, we identified a distinctive population of hemo-vascular precursors which require S1P1 to exert their functions and are essential for cardiac morphogenesis.

Despite its critical role in heart development, the origin of the cardiac endothelium remains intricate. In fact, the heart possesses two different types of endothelium: the endocardium, a structurally and functionally specialized endothelium, which may originate from both vascular endothelium and multipotent cardiac progenitors^1^, and the coronary vascular endothelial cells (CVECs) that seem to develop from endocardium, sinus venous endothelial cells (SVECs) and pro-epicardial precursors^2^,^3^. Notably, some of these progenitors may possess important hemogenic properties. In this regard, blood islands thought to derive *in situ* from hemogenic endothelium have been found in the sub-epicardium and out flow tract endothelium of the embryonic heart^4^,^5^. Another study has shown that *Nkx2.5*+ endocardium contributes to transient definitive hematopoiesis in the early embryonic heart^6^. Collectively, these data support the notion that both endocardium and CVECs may have hemogenic functions during early development and open the possibility that the early embryonic heart may undergo hemo-vasculogenesis, a process whereby the formation of a new blood vessel occurs simultaneously with the generation of blood precursors^7^. However, the origin of the hemogenic endocardium and CVECs is unknown.

The stem cell leukemia *(SCL/Tal1)* gene encodes for a helix-loop-helix transcription factor required for the development of hemangioblasts, the putative common precursor of blood cells and endothelium^8^,^9^. SCL/Tal1 possesses two tissue specific enhancers that direct gene expression either to endothelium or to hematopoietic precursors^9^. Fate tracing studies in zebra fish and expression analysis in mice showed that the SCL gene is expressed in early endocardial precursors and regulates endocardial/myocardial specification and migration of endocardial cells^10^,^11^,^12^. Using mice with tamoxifen inducible *cre recombinase* expression driven by either enhancer^13^,^14^, we recently identified in the early embryonic kidney a *SCL*+ precursor that gives rise to both vascular endothelium and blood cells and requires sphingosine-1-phophate receptor 1 (S1P1) for the development of a normal renal vasculature^15^.

The sphingosine-1-phophate is a bioactive sphingolipid that regulates cell proliferation, migration and stabilization through activation of one of five G protein coupled receptors (S1P1- S1P5). During heart development, S1P1 is predominantly expressed in the endocardium, cardiomyocytes and vascular ECs from E8.5, before it is expressed in the vasculature of the head and body which starts at E10.5^16^,^17^,^18^. Global knockout of *S1P1* in the mouse leads to early embryonic lethality due to vascular abnormalities and impaired heart development^17^. Numerous cardiac cell types respond to the S1P-S1P1 signaling pathway, however, the specific role of S1P1 in each cell type has not been fully examined. A recent study showed that deletion of *S1P1* from cardiomyocytes leads to decreased number of cardiomyocytes in the ventricular compact layer^19^. Interestingly, while investigating the role of S1P1 in the development of the kidney vasculature we found that deletion of *S1P1* in *SCL*+ precursors led to bradycardia, suggesting an indispensable role of S1P1 in *SCL*+ progenitors during heart development^15^. However, in those studies the deletion of *S1P1* was timed to study the developing kidney, which in the mouse starts to form 3 days later than the heart. Therefore, the contribution of *SCL*+ precursors to heart morphogenesis and the role of endothelial *S1P1* in heart development could not be fully assessed. Using *in vivo* and *in vitro* genetic lineage tracings, colony forming assays and a novel crosstransplantation method combined with the inducible and highly specific ablation of *SCL*+ precursors, we tested the hypothesis that those precursors are necessary and require S1P1 for cardiac morphogenesis.

Results show that SCL expression driven by its 5′ end endothelial enhancer (SCL^EC^) labels cardiac hemo-vascular precursors that give rise to both endocardium and CVECs, a subset of which possesses hemogenic potential. Partial and complete ablation of these precursors led to growth retardation, cardiac hypotrophy and loss of endocardium. Further, we induced early deletion (E7.5) of *S1P1* from *SCL*^*EC*^+ precursors and their progeny in mice, which developed non-compaction of both ventricles and abnormal coronary vessels, suggesting an indispensible role of endothelial S1P1 for heart development.

## Results

### *SCL* expression marks cardiac precursors committed to endothelial fate

**Figure 1:**
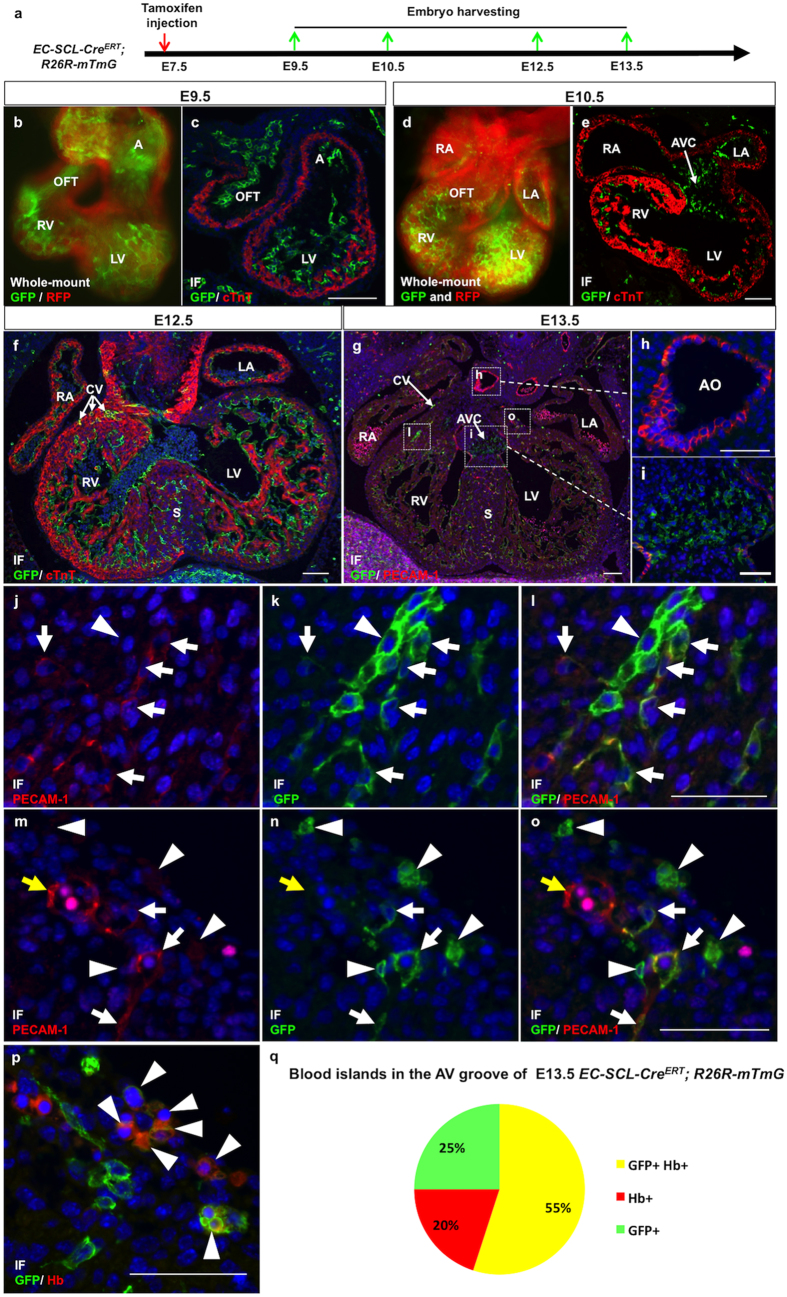
*SCL*^*EC*^+ precursors differentiate into endothelium and endocardium. (**a**) To trace the fate of *SCL*^*EC*^+ precursors, *EC-SCL-Cre-ERT*^+/−^*;R26*^*mTmG*/−^ mice were treated with tamoxifen at E7.5 by maternal injection (red arrow) Embryos were harvested at E9.5, E10.5. E12.5 and E13.5 (green arrows). (**b**–**e**) Whole-mount fluorescent images (**b** and **d**) and immunofluorescence (IF) for GFP (green) and cTnT (red) on heart sections (**c** and **e**) of *EC-SCL-Cre-ERT*^+/−^*;R26*^*mTmG*/−^ mice show that the *SCL*^*EC*^+ precursors give rise to endocardium but not myocardium in both ventricles, atria and outflow tract (OFT) of the heart tube at E9.5 (**b** and **c**) and to the endocardium in the four chambers, atrial-ventricular cushion (AVC) and OFT of the heart at E10.5 (**d** and **e**). (**f**) In addition to endocardium, the *SCL*^*EC*^+ precursors give rise to coronary vascular (CV) endothelial cells (ECs) in the atrial-ventricular groove and in the septum. (**g**–**i**) IF for GFP and PECAM-1 on E13.5 heart sections show that GFP is expressed in all the PECAM-1+ endocardium in the four heart chambers (**g**) and PECAM-1 negative AVC (i), but only in few aortic ECs (**h**).(**j**–**o**) Most of the ECs in the E13.5 heart sections co-expressed GFP and PECAM-1 (arrows). Arrowheads indicate a sub population of GFP+/PECAM-1+ ECs forming a vascular plexus (**j**–**l**) and blood island-like structures (**m**–**o**). Only a few GFP-/PECAM-1+ CV ECs were identified. (yellow arrow). (**p**) IF showed that the blood island-like structures were GFP+/Hb+ (arrowheads).(**q**) Frequency of GFP+ only, Hb+ only and GFP+/Hb+ blood islands in the AVC of E13.5 *EC-SCL-Cre-ERT*^+/−^*;R26*^*mTmG*/−^ mice.A, atria; LV, left ventricle; RV, right ventricle; OFT, outflow tract; AVC, atrial-ventricular cushion; CV, coronary vessel; Ao, aorta; S, septum.Scale bars: 100 μm (**b**–**i**); 50 μm (**j**–**p**).

To investigate the presence and fate of *SCL*^*EC*^+ precursors in the developing heart, first we crossed *EC-SCL-Cre-ERT*^+/−^ mice to *R26*^*mTmG/mTmG*^ mice to obtain *EC-SCL-Cre-ERT*^+/−^*;R26*^*mTmG*/+^ embryos, and performed a single maternal injection of tamoxifen to pregnant mice at embryonic day 7.5 (E7.5), when the cardiac crescent starts to form. Figure 1a shows histological analysis of embryos from E9.5 to E13.5. At E9.5, GFP reporter expression labeled the endocardium of the outflow tract, ventricles and atria but not the myocardium (Fig. 1b and c). At E10.5, GFP labeled the endocardium and the atrioventricular cushions (AVC), which subsequently undergo endothelial-mesenchymal transformation to form the future valves and septa (Fig. 1d and e). From E12.5 to E13.5, GFP expression expanded in the developing endocardium, AVC and coronary vascular endothelial cells (CVECs), and appeared within the ventricular septum (Fig. 1f–i).

Further, double immunofluorescence staining for GFP and the endothelial marker PECAM-1 demonstrated that the endocardium (Fig. 1g) and the majority of CVEC (Fig. 1j–o) co-expressed GFP and PECAM-1. Notably, a small population of CVECs and most of the aortic ECs were not labeled by GFP expression (Fig. 1h and m–o), which may due to either escaping tamoxifen induction or a distinct origin from non *SCL*^*EC*^+ precursors. In addition, we identified GFP+ PECAM-1- cells, which form vascular plexuses in the myocardium (Fig. 1j–l) and 55% of blood-islands co-expressing GFP and hemoglobin (Hb) in the atrioventricular groove (Fig. 1p,q), indicating the immaturity and high mobility of these cells. In summary, the *SCL*^*EC*^+ precursors give rise to endocardium, AVC and the majority of CVECs and blood precursors.

### Embryonic cardiac progenitors possess hemogenic potential

**Figure 2:**
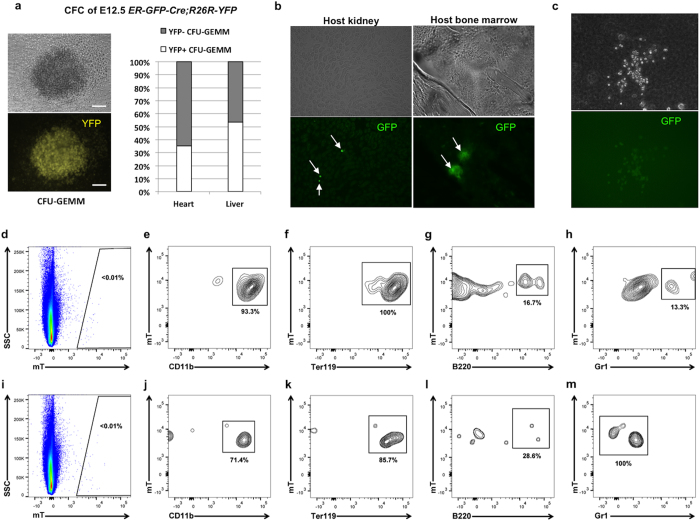
Embryonic Cardiac Progenitors possess Hemogenic Potential. (**a**) Colony Forming Cell assay of cells from E12.5 hearts and livers of *ER-GFP-Cre*^+/−^*; R26*^*YFP*/−^ mice. Representative photograph of a YFP+ CFU-GEMM grown from heart cells. Chart shows the percentage of YFP+ CFU-GEMM from hearts (n = 3) and livers (n = 3). (**b**) In kidney (left) and bone marrow (right) sections of host mice, GFP+ cells (arrows) from the cross-transplanted E9.5 hearts of *ER-GFP-Cre*^+/−^*;R26*^*mTmG*/−^ mice were identified. (**c**) GFP+ CFU formed from bone marrow cells of a host mice transplanted with E9.5 hearts of *ER-GFP-Cre*^+/−^*;R26*^*mTmG*/−^ mice. (**d**–**m**) Flow cytometric analysis of a wild type mouse following irradiation and transplant with mT+ embryonic cardiac cells. (**d**) The spleen of the transplanted host has rare mT+ transplanted cells. (**e**–**h**) These mT+ cells express CD11b and Ter119, but not B220 or Gr1. (**i**–**m**) The bone marrow of the transplanted host had similar numbers of mT+ cells which again express CD11b and Ter119, but not B220 or Gr1.

To investigate the presence of erythroid progenitors in the early embryonic heart, first we crossed *ER-GFP-Cre* mice^20^, harboring a *GFPcre* knockin into the erythropoietin receptor locus, to *R26*^*YFP/YFP*^ and *R26*^*mTmG/mTmG*^ reporter mice. *In vitro* methylcellulose based colony forming unit assays using cells from E12.5 embryonic hearts and livers of *ER-GFP-Cre*;*R26*^*YFP/YFP*^ mice showed that cells from these organs formed two types of early stage HSC colonies: CFU-GEMM colonies, containing granulocytes, erythrocytes, monocytes/macrophages and megakaryocytes and CFU-GM colonies, containing granulocytes and macrophages. Among those colonies, the YFP reporter was only found homogeneously expressed in 35% of the CFU-GEMM colonies derived from heart cells (Fig. 2a). These results indicated the existence of at least two different early hematopoietic precursors within the embryonic heart.

To further assess the hemogenic capacity of the embryonic heart we transplanted E9.5 hearts (n = 10) from *ER-GFP-Cre*;*R26*^*mTmG*^ mice under the kidney capsule of adult wild type host mice (n = 5) for 5 days. The transplanted hearts grew and were beating at the time of harvest (Movie 1). GFP+ cells originating from the transplanted hearts were observed within the host kidney as isolated cells at a rate of 5 cells/cm^2^ and in the host bone marrow not only as single isolated cells (46 cells/cm^2^) but also as colonies of >15 cells (22 colonies/cm^2^) (Fig. 2b), suggesting these precursors originating from the transplanted embryonic hearts can home to, proliferate in the bone marrow and contribute to the circulation. Furthermore, *in vitro* methylcellulose based colony forming unit assays grown from bone marrow cells of the host mice developed GFP+ colonies at a rate of 1 GFP+ colony/100 colonies originating from the transplanted hearts (Fig. 2c). In order to extend the findings of progenitors with hematopoietic potential within the embryonic heart, we isolated single cells from E9.5 hearts (n = 6) of *R26*^*mTmG/mTmG*^ mice (where all nucleated cells express mTomato) and injected then into the tail vein of an irradiated wild type mouse. Four weeks later, we found mTomato+ (mT+) cells in the bone marrow, spleen, and peripheral blood of the host mouse. We characterized the immunophenotype of these cells using flow cytometry, and although these cells were rare (<0.01% of nucleated spleen and marrow cells), they express hematopoietic markers (Fig. 2d–m). Consistent with our prior colony assays, mT+ cells isolated from embryonic hearts and transplanted into a recipient mouse express the cell surface markers CD11b and Ter119, markers of monocytes and erythrocytes respectively but were negative for B cell (B220) and granulocyte (Gr1) markers. This transplant experiment confirms the presence of cardiac progenitors with hemogenic potential.

**Figure 3:**
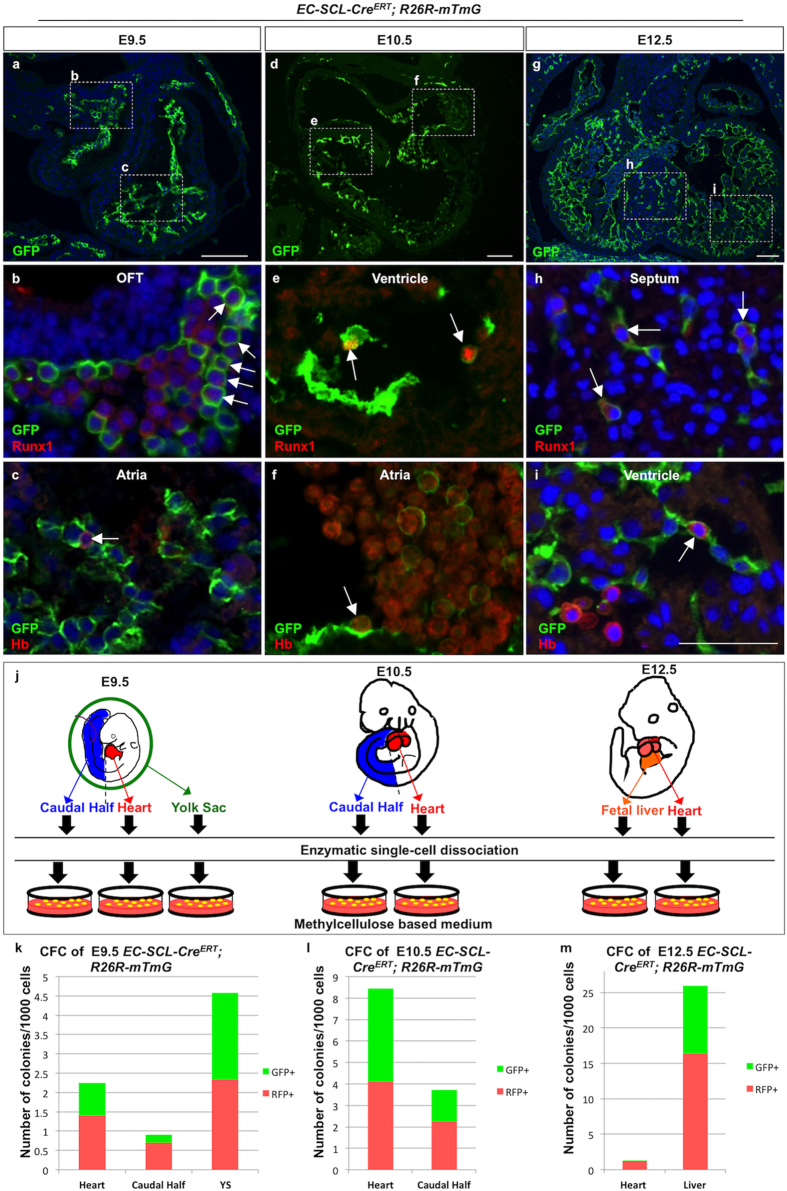
*SCL*^*EC*^+ precursors contribute to hematopoiesis in the early embryonic heart. (**a**–**i**) IF for GFP, Runx1 and hemoglobin (Hb) on sections of hearts of *EC-SCL-Cre-ERT*^+/−^*;R26*^*mTmG*/−^ mice at E9.5 (**a**–**c**), E10.5 (**d**–**f**) and E12.5 (**g**–**i**). a, d and g show IF for GFP in the heart sections at low magnification. (**b**,**e** and **h**) show GFP+/Runx1+ cells in the OFT at E9.5 (**b**, arrows), ventricular endocardium at E10.5 (**e**, arrows) and in the septal CVECs at E12.5 (h, arrows) at high power views of the boxed area in (**a**,**d** and **g**), respectively. (**c**) f and i show GFP+/Hb+ cells in the atrial endocardium at E9.5 and E10.5 (**c** and **f**, arrows) and in ventricular endocardium at E12.5 (**i**, arrow) at high magnification. (**j**) Schematic of colony forming-cells (CFC) assays using cells from whole hearts (red), caudal half (blue), yolk sac (green) and fetal liver (orange) from E9.5, E10.5 and E12.5 embryos. (**k**–**m**) Bar graph shows the number of GFP+ (green) and RFP+ (red) CFU-Mix colonies that grew from the cells of hearts and other hematopoietic organs from E9.5 (**k**, n = 3), E10.5 (l, n = 4) and E12.5 (**m**, n = 5) *EC-SCL-Cre-ERT*^+/−^*;R26*^*mTmG*/+^ mice treated with tamoxifen at E7.5. YS, yolk sac. Scale bar: 100 μm (**a**,**d** and **g**) 50 μm (**b**,**c**,**e**–**f** and **h**–**i**).

Next, to evaluate whether in the developing heart *SCL*^*EC*^+ precursors possess intrinsic hemogenic properties we performed the following series of experiments. Immunofluorescence for GFP, Runx1 (a master regulator of blood formation) and Hb in heart sections of *EC-SCL-Cre-ERT*^*Cre*/−^*;R26*^*mTmG*/−^ mice (following the scheme in Fig. 1a) showed at E9.5 and E10.5 cells co-expressing GFP and Runx1 or Hb in the endocardium of the outflow tract (OFT), ventricles and atria (Fig. 3a–f). At E12.5 (Fig. 3g) we observed GFP+/Runx1+ blood islands in the ventricular septum (Fig. 3h) and GFP+/Hb+ cells in ventricular endocardium (Fig. 3i). These data suggest that within the developing embryonic heart *SCL*^*EC*^+ precursors give rise to hemogenic endocardium.

To further assess the hemogenic capacity of the early *SCL*^*EC*^+ precursors in the embryonic heart, we performed methylcellulose-based colony forming cell (CFC) assays. We harvested cells from the hearts and different embryonic areas including major hematopoietic organs (yolk sac and caudal half at E9.5, caudal half at E10.5 and fetal liver at E12.5), at different embryonic stages from *EC-SCL-Cre-ERT*^*Cre*/−^*;R26*^*mTmG*/−^ mice with tamoxifen treatment at E7.5 (Fig. 3j). Similar to the results of cells grown from hematopoietic organs, two types of early stage hematopoietic colonies: CFU-GEMM (granulocytes, erythrocytes, monocytes/macrophages and megakaryocytes) and CFU-GM (granulocytes and macrophages) formed from the cells of the hearts. The CFU-Mix (GEMM and GM) colonies formed at a rate of 2.3 (±1) per 1000 heart cells with 43% GFP expressing colonies at E9.5 (Fig. 3k), reached a peak at E10.5 (8.4 ± 0.6 colonies per 1000 cells, 50% GFP+) (Fig. 3l) and by E12.5 decreased to 1.3 (±0.3) per 1000 cells with 8.4% GFP+ colonies (Fig. 3m). Overall, these results indicated that the *SCL*^*EC*^+ precursors give rise to early hematopoietic precursors within the early embryonic heart in a developmentally regulated manner.

### Ablation of *SCL* ^*EC*^ + precursors impairs hemo-vasculogenesis and heart development

**Figure 4:**
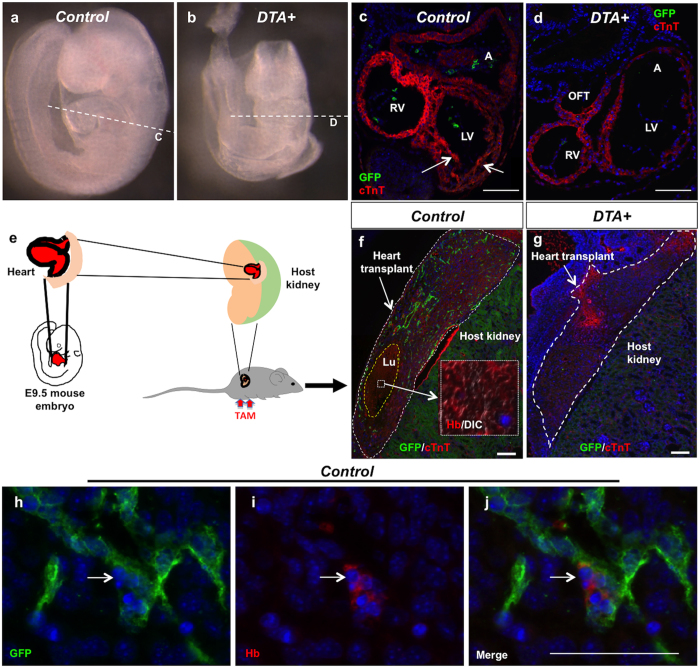
Ablation of *SCL*^*EC*^+ precursors impairs hemo-vasculogenesis and heart development. (**a**,**b**) Low-dose of tamoxifen injected at E7.5 led to delayed growth of *DTA*+ (*EC-SCL-Cre-ERT*^+/−^*; R26*^*DTA*/−^*;R26*^*mTmG*/−^) mice at E9.5(**b**), whereas control embryos (*EC-SCL-Cre-ERT*^+/−^*;R26*^*mTmG*/−^) did not display any abnormality (**a**). Dashed lines indicate the position of the heart sections shown in c and d. (**c**,**d**) IF for GFP (green) and cTnT (red) show that control mice developed a normal heart with a subset of the endocardium labeled by GFP expression. (**c**) GFP+ cells were absent in DTA+ mice. (**e**) Schematic of embryonic heart transplantation. The whole heart from E9.5 mouse embryos was dissected and transplanted under the kidney capsule of an adult wild type host mouse. After surgery, the host mouse was injected with tamoxifen to induce cre expression with subsequent DTA and reporter expression. The transplanted hearts and host kidneys were harvested 7 days after surgery. (**f**–**g**) IF for GFP (green) and cTnT (red) show that the control transplanted heart developed GFP+ endocardium (**f**, within the white dashed line area). The yellow dashed line indicate a lumen (Lu), filled with Hb+ blood cells (in red, shown in the inset). The DTA+ transplanted heart completely lost the lumen structure and GFP+ cells. (**g**, white dashed line). (**h**–**j**) IF for GFP and Hb show a GFP+/Hb+ blood island in the section of the control transplanted heart (arrows). Scale bars: 100 μm (**c**,**d**,**f**,**g**); 50 μm (**h**–**j**).

To investigate whether *SCL*^*EC*^ + precursors were indispensable for the normal development of the heart we designed the following ablation experiment that involves the expression of diphtheria toxin fragment A (DTA). We crossed *R26*^*DTA/DTA*^ mice with *EC-SCL-Cre-ERT*^+/−^ *and R26*^*mTmG/mTmG*^ mice to generate *EC-SCL-Cre-ERT*^+/−^*;R26*^*DTA*/+^*;R26*^*mTmG*/+^ (*DTA*+) mice, which express both DTA and GFP in the *SCL*^*EC*^+ precursors. All the *DTA*+ embryos died within 24hrs with full dose of tamoxifen injection (1 mg/30 g bodyweight) at E7.5, whereas their *Cre*+ (*EC-SCL-Cre-ERT*^+/−^*;R26*^*mTmG*/+^) control littermates were normal (data not shown). When we injected pregnant mice with half the dose of tamoxifen (0.5 mg/30 g bodyweight) to partially ablate the *SCL*^*EC*^+ precursors, the *DTA*+ embryos survived to E9.5 but showed significant growth retardation (Fig. 4a and b). Immunofluorescence for GFP and cTnT on heart sections of control embryos further confirmed that part of the *SCL*^*EC*^+ lineage endocardium was still labeled by GFP+ expression, whereas the *DTA*+ embryos almost completely lost the *SCL*^*EC*^+ lineage endocardium labeled with GFP. Additionally, the *DTA*+ embryos developed thin myocardium with impaired trabeculation (Fig. 4c and d) suggesting the requirement of *SCL*^*EC*^+ precursors for the normal development of the heart.

Because the *SCL*^*EC*^+ precursors contribute to the vascular system all over the embryo, the heart developmental abnormities observed in the *DTA*+ embryos may be influenced by concomitant systemic vascular abnormalities. To investigate whether ablation of *SCL*^*EC*^+ precursors affects cardiac development intrinsically, we developed an *ex vivo* cross-transplantation model, which allows the development of the embryonic heart under the kidney capsule of the host mice and the visualization of a beating heart at the time of harvesting (Movie 1). After transplantation of hearts from E9.5 *DTA*+ mice and their control siblings under the kidney capsule of wild type adult host mice we induced DTA and GFP expression in *SCL*^*EC*^+ precursors by intraperitoneal tamoxifen injections in the host mice (Fig. 4e). Immunofluorescence staining for GFP and cTnT showed that the GFP+ cells were present within the heart of the control transplants (Fig. 4f), with GFP+/Hb+ blood islands (Fig. 4h–j and Supplemental Fig. 1), which further confirmed the contribution of *SCL*^*EC*^+ precursors to intrinsic hemo-vasculogenesis in the heart. Interestingly, the control transplanted hearts developed lumens filled by red blood cells. But no GFP+ cell was found lining in the heart lumen suggesting that the expression of SCL may have occurred previous to the induction of cre by tamoxifen administration after transplantation. However, the transplanted hearts from *DTA*+ mice did not beat and completely lost the GFP+ cells and the heart lumen structure (Fig. 4g). These experiments underscore the requirement of *SCL*^*EC*^+ precursors within the early embryonic heart for hemo-vasculogenesis and organogenesis.

### *S1P1* expressed in *SCL* ^*EC*^ lineage endothelium regulates heart development

Recently, we reported that timed (from E10.5) conditional deletion of *S1P1* from *SCL*+ ECs results in abnormal heart development^15^. To fully characterize the role of endothelial S1P1 during early cardiac development, we treated *cEC-SCL-S1P1KO* mice and their control siblings with tamoxifen at E7.5 to induce GFP reporter expression and/or *S1P1* deletion in the *SCL*^*EC*^+ lineage cells. We observed embryonic lethality around E14.5. Before E13.5, the littermate controls of the *cEC-SCL-S1P1KO* embryos were morphologically normal, indistinguishable from non-injected ones. Therefore, we mainly characterized the hearts of *cEC-SCL-S1P1KO* mice from E12.5 to E13.5.

**Figure 5:**
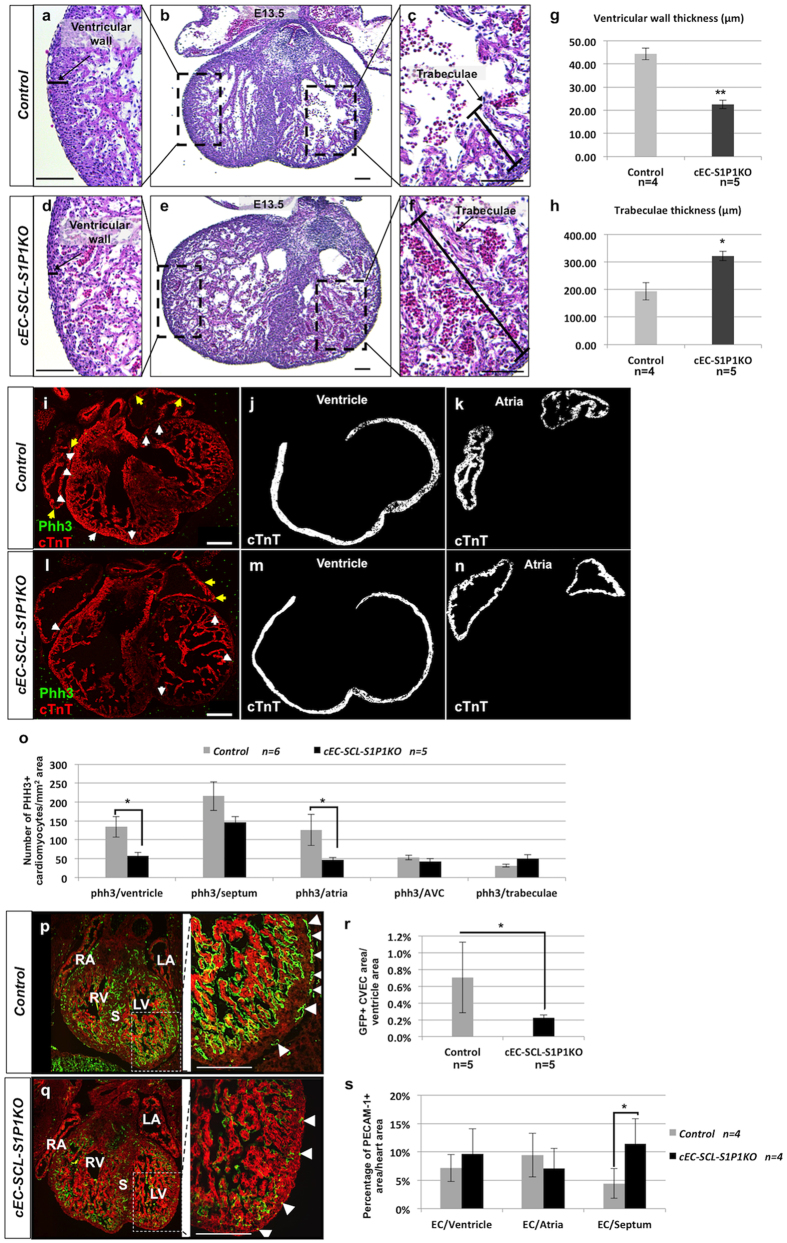
*S1P1* in *SCL*^*EC*^+ precursors is crucial for normal heart development. (**a**–**f**) Hematoxylin and eosin (H&E) staining of heart sections from E13.5 *control (EC-SCL-Cre-ERT*^+/−^*;S1P1*^*fl*/+^*;R26*^*mTmG*/−^) (**a**–**c**) and *cEC-SCL-S1P1KO (EC-SCL-Cre-ERT*^+/−^*;S1P1*^*fl*/−^*;R26*^*mTmG*/−^) (**d**–**f**) mice show thinner ventricular wall (**d**) and thicker trabeculae (**f**) in *cEC-SCL-S1P1KO* mice when compared to *control* mice (**a** and **c**). (**g**) Measurements of the thickness of ventricular compact layer show thinner ventricular wall of the hearts from E12.5 *cEC-SCL-S1P1KO (EC-SCL-Cre-ERT*^+/−^*;S1P1*^*fl*/−^*;R26*^*mTmG*/−^) mice (n = 4) than those from control (*EC-SCL-Cre-ERT*^+/−^*;S1P1*^*fl*/+^*;R26*^*mTmG*/−^) mice (n = 5). **p < 0.01. (**h**) Measurements of the thickness of trabecular tissue show thicker trabeculae in hearts from E12.5 *cEC-SCL-S1P1KO (EC-SCL-Cre-ERT*^+/−^*;S1P1*^*fl*/−^*;R26*^*mTmG*/−^) mice (n = 4) than in those from control (*EC-SCL-Cre-ERT*^+/−^*;S1P1*^*fl*/+^*;R26*^*mTmG*/−^) mice (n = 5). *p < 0.05. (**i**–**n**) i and l show IF for phosphorylated histone H3 (phh3) (in green) and cTnT (in red) in the heart sections of E12.5 *control* (i) and *cEC-SCL-S1P1KO* (**l**) mice. Yellow arrows show phh3+cTnT+ cells in the atria. White arrows show phh3+cTnT+ cells in the ventricular area. j, k, m and n show defined ventricular (**j** and **m**) and atria area (k and n) using imageJ. (**o**) Quantification of the number of proliferating phosphorylated histone H3 (phh3)+cTnT+ cells in different cardiac structures of E12.5 *control* (n = 6) and *cEC-SCL-S1P1KO* (n = 5) mice. The *cEC-SCL-S1P1KO* hearts showed significantly reduced proliferation in the myocardium of ventricles and atria, but not in the septum, AVC or trabeculae. *p < 0.05. (**p**,**q**) Fluorescent images of heart sections from control (*EC-SCL-Cre-ERT*^+/−^*;S1P1*^*fl*/+^*;R26*^*mTmG*/−^) and *cEC-SCL-S1P1KO (EC-SCL-Cre-ERT*^+/−^*;S1P1*^*fl*/−^*;R26*^*mTmG*/−^) mice. *SCL*^*EC*+^ cells express GFP whereas all other cells express RFP. Pictures on the right side show the white dashed squares at higher magnification. Comparing to the heart from *control* mice (**p**, right), the heart of *cEC-SCL-S1P1KO* mice (**q**, right) show fewer ventricular coronary vessels (arrowheads). RA, right atria; LA, left atria; RV, right ventricle; LV, left ventricle. (**r**) Measurement of the area of GFP+ ventricular CVECs/ventriclular compact area in heart sections of E12.5 *control* (n = 5) and *cEC-SCL-S1P1KO* (n = 5) mice showed less CVECs in the *cEC-SCL-S1P1KO* heart than the control heart.*p < 0.05. (**s**) Measurement of the area of PECAM-1+ cells in different structures of hearts from E12.5 *control* (n = 4) and *cEC-SCL-S1P1KO* (n = 4) mice show increased septal ECs in the *cEC-SCL-S1P1KO* heart. The septums are shown in the lined area in Supplemental Figure 2g–j. PECAM-1+ areas within the septum were measured. There were no differences in the endocardium of ventricles and atria. *p < 0.05. Scale bars: 100 μm (**a**–**f**); 200 μm (**i**–**n** and **p**,**q**).

The *cEC-SCL-S1P1KO* mice developed thin ventricular walls (22.6 ± 1.8 μm, versus 44.4 ± 2.5 μm in control mice p < 0.01) and thick trabeculae (321.8 ± 16.5 μm, versus 193.7 ± 31.2 μm in control mice p < 0.05) (Fig. 5a–h). To study whether proliferation of the myocardium was affected, we performed co-immunofluorescence staining for phosphorylated histone H3 (phh3) and the myocardial marker cardiac troponin (cTnT) on heart sections of E12.5 *cEC-SCL-S1P1KO* and control mice (Fig. 5i–n). The results demonstrated a remarkable decrease in the proliferation of the myocardium in both ventricles and atria of the *cEC-SCL-S1P1KO* mice (Fig. 5i–o). However, there was no significant difference in the trabeculae (Fig. 5o).

Interestingly, we found that *cEC-SCL-S1P1KO* embryos developed less peri-truncal CVECs within the ventricular wall than the controls (Fig. 5p,q, and r, Supplemental Figure 2a–f), whereas their CVECs in the septum were increased (Fig. 5s, Supplemental Fig. 2g–j). We did not find a difference in the number of PECAM-1+ cells within the endocardium or atria between the *cEC-SCL-S1P1KO* and control embryos (Fig. 5s).

## Discussion

In this study we identified cardiac precursors that are committed to an endothelial fate and give rise to endocardium and CVECs. A subset of those precursors undergo hemo-vasculogenesis to generate ECs and blood cells. Genetic ablation of these precursors *in vivo* in the whole embryo or in embryonic heart transplants resulted in absence of endocardium and subsequent abnormal myocardial development. Further, we specifically deleted *S1P1* from cardiac endothelium during early heart development and revealed essential roles of endothelial S1P1 for the development of ventricular and atrial cardiomyocytes and coronary vascular formation.

Collective data from lineage analysis suggested that the endocardium, CVECs and vascular endothelium elsewhere are highly related in cellular origin and gene expression profile. For instance, fate-tracing studies of *Nfatc1*+ endocardium demonstrated that the endocardium is a major source of the arterial CVECs through angiogenesis^3^. Albeit the majority of venous CVECs and a small number of arterial CVECs were derived from the dedifferentiated sinus venous endothelium^21^. In addition, another study has identified a subset of pro-epicardial precursors giving rise to a small population of CVECs and smooth muscle cells^2^. However, none of the marker genes used in those studies exclusively labeled the whole population of cardiac ECs, including endocardium and CVECs. The lack of specific markers for intermediate precursors committed to cardiac endothelial fate has hampered our understanding of the molecular basis of cell fate decision and cell differentiation in the heart. Here, we report that time-labeled early *SCL*^*EC*^+ precursors give rise to all the endocardium and the majority of CVECs during heart development, which will enable the isolation of early cardiac EC precursors for analyzing genetic networks underling cardiac development and will ultimately be of help for the cardiac regeneration field.

Our studies identified in the embryonic heart intrinsic *SCL*^*EC*^+ precursors that differentiate not only into endothelium and endocardium but also have hemogenic capacity. Using lineage tracing strategies, *SCL*^*EC*^+ precursors are found in blood islands of the developing heart, express hematopoietic markers including Runx1 and Hb, contribute to circulating blood nucleated cells and give rise to erythroid and myeloid colonies in culture indicating that they are multipotent myeloid precursors. The CFC studies showed that fewer colonies grew from the E12.5 heart than from earlier stages, being E10.5 at its peak. This may due to low percentage of endothelium in the whole heart, since the cardiomyocytes and other types of cells proliferate quickly and account for a large proportion of the total heart cell mass at this age. Further, the lower percentage of GFP expressing colonies from the E12.5 heart suggested a decreased contribution of *SCL*^*EC*^+ hemogenic precursors at this later stage which is in agreement with the transient hemogenic function of the embryonic heart previously described^6^. Although, cell surface marker expression studies in embryonic stem cells suggested that cardiac progenitors and hemangioblasts were two distinct populations^22^,^23^,^24^,^25^, our data suggest a contribution of cardiac hemo-vascular precursors to the hemogenic endocardium and CVECs.

Further, *in vivo* experiments of embryonic heart cells transplanted into adult recipient mice, both as heart explants under the host kidney capsule and as single cells injected into the tail vein of an irradiated host, demonstrated that cells from the embryonic heart can home to the bone marrow, differentiate into myeloid and erythroid lineages and access the main circulation. Whether *SCL*^*EC*^+ precursors differentiate into hematopoietic stem cells and therefore also give rise to lymphoid lineages remains to be determined. Furthermore, to evaluate if *SCL*^*EC*^+ cells are the sole heart progenitors with embryonic hemogenic activity or other non-*SCL*^*EC*^ cells within the developing heart share the same ability will require additional lineage tracing studies utilizing different cre transgenic mouse lines.

Here we described a novel cross-transplantation approach in which the embryonic heart is placed under the kidney capsule of an adult wild type mouse. Under these conditions, the developing heart continues to undergo morphogenesis, contracts rhythmically and develops its normal cardiac cavities. On the other hand, selective induced ablation of *SCL*^*EC*^+ precursors in similarly transplanted embryonic hearts, results in the loss of *SCL*^*EC*^+ derived fluorescent cells, lack of cavity formation and loss of cardiac contractility indicating the crucial role of these precursors during heart development.

The lack of GFP+ cells lining the lumens of transplanted *EC-SCL-Cre-ERT*^+/−^*;R26*^*mTmG*/+^ hearts may be due to late tamoxifen induction (at E9.5 after transplantation), since in our lineage studies we started to label the endocardium by injecting tamoxifen at E7.5. It is also possible that the environment in the transplantation affected the development of endocardium within the heart lumens. We have previously observed that a similar cross-transplantation approach for the prevascular embryonic kidney not only allows nephrogenesis to occur but also the development of the kidney vasculature with -normal- distribution and differentiation of the cells that compose the kidney arterioles^15^,^26^. Further characterization of the development of the embryonic heart using this cross-transplantation model is underway.

Equally important, our *cEC-SCL-S1P1KO* mice developed thin ventricular myocardium accompanied with abnormal trabeculation and coronary vascular development, which mimics ventricular non-compaction cardiomyopathy observed in human patients. Given that the cause of this disease is still elusive, it would be important to determine whether mutations of the *S1P1* receptor are found in patients with non-compaction cardiomyopathy^27^. Similar to the *S1P1* global knockout mice^17^, our *cEC-SCL-S1P1KO* mice showed reduced myocardial proliferation in the ventricles and atria, suggesting that myocardial development is regulated by endothelial S1P1 in a non-autonomous manner. Although we found thickened trabecular tissue in the heart of *cEC-SCL-S1P1KO* mice, their proliferation was not significantly increased. It has been reported that the trabeculation is regulated by growth factors secreted by the endocardium^28^. Therefore, deletion of S1P1 from endocardium may affect expressions of these factors and consequently result in abnormal trabeculae. Notably, the coronary vessels in the ventricle wall and septum showed different phenotype in response to deletion of endothelial *S1P1*, which suggests a potentially distinct role of S1P1 during their development. It is known that coronary vascular development is regulated by growth factors secreted by both epicardium and myocardium^29^. Hence, different signaling pathways (such as VEGF, Angiopoietin and FGF) may regulate the development of CVECs in the ventricular wall and septum. Whether S1P1 interacts with those signaling pathways to regulate coronary vascular development needs to be studied. We have previously found that deletion of endothelial *S1P1* results in increased proliferation of ECs in the kidney. Whereas during cardiac development S1P1 may also prevent excessive proliferation of ECs in the septum, in the coronary vessels seems to have an opposite effect.

In summary, in this study we identified an indispensable common precursor labeled by *SCL*^*EC*^ expression for all the cardiac endothelium, a subset of which possesses hemogenic potential. Furthermore, we demonstrated that endothelial S1P1 is crucial for myocardial and coronary vascular development. These data and the mouse models we generated may prove useful to understand genetic defects and signaling pathways linked to congenital heart diseases.

## Methods

### Animals

For fate tracing studies, colony-forming cell assays and embryonic heart transplantations, constitutive *ER-GFP-Cre*^20^ and tamoxifen inducible *EC-SCL-Cre-ERT* mice^13^ were crossed to B6.129(Cg)*-Gt(ROSA)26Sor*^*tm4(ACTB-tdTomato,-EGFP)Luo*^*/*J (*R26*^*mTmG/mTmG*^)^30^ or B6.129 × 1-Gt(ROSA)26Sor^tm1(EYFP)Cos/J^ (*R26*^*YFP/YFP*^)^31^ reporter mice. Pregnant mice from the tamoxifen inducible *EC-SCL-Cre-ERT* crosses were injected with tamoxifen intraperitoneally at a dose of 1 mg/30 g of body weight. To genetically ablate *SCL*^*EC*^+ precursors, *EC-SCL-Cre-ERT* mice were crossed to B6.129-*GT(ROSA)26SSor*^*tm1(DTA)Lky*^*/*J mice (*R26*^*DTA/DTA*^)^32^ and *R26*^*mTmG/mTmG*^ mice. Partial ablation of *SCL*^*EC*^+ precursors was achieved by injecting pregnant mice with half dose of tamoxifen (0.5 mg/30 g of body weight). To study the role of S1P1 in cells from the *SCL*^*EC*^+ lineage during heart development, *cEC-SCL-S1P1KO* mice were generated as previously described^15^. Wild type mice (3–5 months old) were used as hosts for transplantation of embryonic hearts or heart cells (the latter after irradiation). All procedures were performed in accordance with the Guiding Principles for Research Involving Animals and Human Beings by the American Physiological Society and were approved by the University of Virginia Animal Care Committee.

### Whole-mount organ imaging and immunofluorescence

For whole-mount heart imaging, the hearts from *EC-SCL-Cre-ERT*^+/−^*;R26*^*mTmG*/+^ mice at E9.5 (n = 3), E10.5 (n = 3) and E12.5 (n = 4) were micro-dissected and fixed in 4% paraformaldehyde for 5 minutes and placed in cold PBS. Images were captured using a Q Imaging RETIGA EXi camera connected to a Leica DMIRE2 microscope.

Immunofluorescence was performed on 5 μm sections of Bouins fixed and paraffin embedded tissues following standard protocols^33^,^34^. Primary antibodies used include anti-GFP (1:500 dilution, Abcam, ab13970), anti-PECAM-1 (1:500 dilution, Santa Cruz, sc1506-R), anti-phh3 (1:200 dilution, Cell Signaling, #9701S), anti-cTnT (1:200 dilution, Abcam, ab8295), anti-Hb (1:500 dilution, DAKO, A0118) and anti-Runx1 (1:500 dilution, Abcam, ab23980). The secondary antibodies used were Alexa Fluor 488 goat anti-chicken IgG (H + L) (GFP), Alexa Fluor 568 donkey anti-rabbit IgG (H + L) (PECAM-1, phh3, Hb and Runx1) and Alexa Fluor 568 donkey anti-mouse IgG (H + L) (1:500 dilution; Life Technologies, Carlsbad, CA). Nuclei were stained with Hoechst 33342 (1:1000dilution, Invitrogen, H3570). Images were obtained using a Leica DFC360 FX digital camera connected to a Leica DFC 480 microscope.

### Colony-forming-cell assays

Hearts and livers at E12.5 from *ER-GFP-Cre*^+/−^; *R26*^*mTmG*/+^ embryos and hearts [at E9.5 (n = 3), E10.5 (n = 4) and E12.5 (n = 5)], caudal halves [at E9.5 (n = 3) and E10.5 (n = 4)], yolk sacs (at E9.5, n = 3) and fetal livers (at E12.5, n = 5) from *EC-SCL-Cre-ERT*^+/−^*;R26*^*mTmG*/+^ embryos were micro-dissected in Iscove’s MDM with 2% FBS and disaggregated with collagenase (M7902 Stemcell technologies, Vancouver, BC, Canada) at 37 °C. The dissociated single cells from each organ were added to 1 ml of Methocult GF (M3434, StemCell Technlogies, Vancouver, BC, Canada) and cultured in 24 well cell culture plates at 37 °C in 5% humidified CO_2._ After 12 days of incubation, the colonies were scored as described previously^15^. Bone marrow cells (1.0 × 10^6^ cells/well) isolated from wild type host mice transplanted with *ER-GFP-Cre;R26*^*mTmG*/+^ hearts were cultured in 6 well plates using Methocult GF (M3434; StemCell Technologies, Vancouver, BC, Canada).

### Embryonic heart transplantation

Whole embryonic hearts from E9.5 *ER-GFP-Cre;R26*^*mTmG*/+^ (n = 10) or *EC-SCL-Cre-ERT*^+/−^*;R26*^*DTA*/+^*;R26*^*mTmG*/+^ mice (*DTA* + , n = 3) and their control siblings (*EC-SCL-Cre-ERT*^+/−^*;R26*^*mTmG*/+^, n = 3) were micro-dissected in 5% FBS medium (DMEM-Ham’s F-12, Cat# 11320) and transplanted under the kidney capsule of adult host mice following similar procedures previously described for embryonic kidneys^15^. Recipient mice were anesthetized with tribromoethanol intraperitoneally at a dose of 250 mg/kg. Ophthalmic ointment was applied to eyes to prevent drying during surgery. The mice were positioned lying with their left side up on a heated operating surface at 37 °C. After hair removal, the animals were aseptically prepped, skin cleaned with povidone iodine and alcohol. Sterile instruments were used throughout the surgical procedure. A left back incision was made along the dorsal lumbar side above the kidney and the muscle layers were dissected to expose the left kidney. Next, a small cut was made in the kidney capsule, and a blunt 20-gauge needle was carefully inserted into the incision to create a 0.8–1 cm subscapular tunnel towards the upper pole of kidney. An embryonic heart was placed (using a forceps) through the tunnel. Additional tunnels were made towards the lower pole and middle area to transplant additional hearts. One to three fetal hearts were placed under the kidney capsule of each host. The left kidney was placed back in the abdominal cavity. The muscle layers and skin were sutured separately. After recovering from the anesthesia on a heating pad at 37 °C, and administration of the analgesic Buprenorphine 0.1–0.2 mg/kg SC q 8–12 hours the mice were returned to the vivarium and monitored for health and fitness. Host mice transplanted with *EC-SCL-Cre-ERT*^+/−^*;R26*^*DTA*/+^*;R26*^*mTmG*/+^ and *EC-SCL-Cre-ERT*^+/−^*;R26*^*mTmG*/+^ embryonic hearts.were injected post surgery with tamoxifen (1 mg/30 g of body weight) intraperitoneally for 7 days. Mice (n = 5) transplanted with *ER-GFP-Cre;R26*^*mTmG*/+^ hearts (n = 10) were studied at 5 days after transplantation. The kidneys with the implanted hearts were removed and fixed by 2–4% PFA solution for immunostaining. Bones and bone marrow from host mice transplanted with *ER-GFP-Cre;R26*^*mTmG*/+^ hearts were processed for histological analysis and colony-forming-cells assays as described above.

### Measurement of the thickness of ventricular wall and trabeculae

To measure the thickness of the ventricular wall and trabeculae, we performed immunofluorescence for the myocardial marker cardiac troponin T (cTnT) in 5 comparable heart sections of E12.5 *cEC-SCL-S1P1KO* (n = 5) and control (n = 4) embryos. As described previously^35^, the ventricular wall thickness was determined by the average length of six lines drawn between the epicardium and the edge of the myocardium compact layer. (Fig. 5a and d) And the thickness of trabeculae was determined by the average length of six lines drawn between the edge of ventricular wall and the tip of trabeculae. Measurements were performed by image J.

### Measurement of myocardium proliferation

To measure the number of proliferating myocardial cells, we performed double-immunofluorescences for phosphorylated histone h3 (phh3) and cTnT in 5 comparable sections from the heart of E12.5 *cEC-SCL-S1P1KO* (n = 5) and control (n = 6) embryos. The number of phh+/cTnT+ cells were counted in each cardiac structure (ventricles, atria, septum, AVC and trabeculae). The areas of each cTnT+ cardiac structure were measured by imageJ.

### Measurement of areas of endocardium and coronary vascular endothelial cells (CVECs)

To measure the area of GFP+ CVECs in the ventricles, we performed double-immunofluorescences for GFP and cTnT on 5 comparable sections from the heart of E12.5 *cEC-SCL-S1P1KO* (n = 5) and control (n = 5) embryos. The GFP+ ventricular CVEC areas and the cTnT+ ventricular area were measured using imageJ (Supplemental Fig. 2a–f). The areas of GFP+ endocardium and septal CVECs were manually excluded. The values were expressed as percentages:


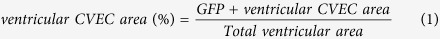


To measure the area of PECAM-1+ endocardium in the ventricles and atria and CVEC in the septum, we performed immunofluorescence for PECAM-1 and cTnT in 5 comparable sections from the heart of E12.5 *cEC-SCL-S1P1KO* (n = 4) and control (n = 4) embryos. The PECAM-1+ areas and cTnT+ areas were measured using imageJ (Supplemental Fig. 2g–j). The values were expressed as percentages:


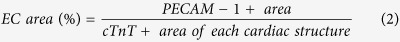


### Transplantation of embryonic heart cells into an irradiated host

E9.5 hearts (n = 6) from *R26*^*mTmG/mTmG*^reporter mice (express fluorescent tomato in every cell) were harvested in 2% FBS in IMDM (Cat#12440–053), and washed several times with 2% FBS in IMDM. After incubation in 0.25% collagenase type I (Cat#07902, Stemcell Technologies) at 37 °C cells were dissociated by gently pipette tissues up and down, every 10–15 min until a single cell suspension was obtained. Cells (2.93 × 10^4^) were resuspended in 100 μl 5% FBS in IMDM and injected intravenously into an irradiated (10 Gy) mouse.

### Flow Cytometry

Bone marrow and spleen of a transplant recipient mouse were harvested, and single cell suspensions were prepared as previously described^34^. Briefly, the femurs and tibias were dissected using sterile technique, flushed with PBS/5% FBS, and cells were passed through a 70 μm strainer. Spleens were dissected cleanly using sterile technique, placed on a 70 μm strainer and using the plunger end of a syringe, gently passed through the strainer. Red blood cells were lysed using RBC lysis buffer (Biolegends, San Diego, California). Cells were resuspended in PBS/5% FBS, and then counted using a Cellometer Mini cell counter (Nexcelom, Lawrence, MA). One million cells of each sample were then distributed into microcentrifuge tubes and labelled with fluorochrome-conjugated antibodies APC/Cy7-B220, FITC-Ter119, PE/Cy7-Gr1, and PerCP/Cy5.5-CD11b (all antibodies were purchased from Biolegends and added at predetermined optimum concentrations). Immunophenotyping was performed in the UVA Flow Cytometry core lab using a Fortessa cytometer, and data were analyzed using the FlowJo program (FlowJo LLC, Ashland, Oregon).

### Statistical analysis

Data are shown as mean ± SEM. Statistical analysis was carried out by Student’s t test. A p < 0.05 was considered significant.

## Additional Information

**How to cite this article:** Hu, Y. *et al*. Identification of cardiac hemo-vascular precursors and their requirement of sphingosine-1-phosphate receptor 1 for heart development. *Sci. Rep.* **7**, 45205; doi: 10.1038/srep45205 (2017).

**Publisher's note:** Springer Nature remains neutral with regard to jurisdictional claims in published maps and institutional affiliations.

## Supplementary Material

Supplementary Movie 1

Supplementary Information
