# Supplementary material for: Identification of cardiac hemo-vascular precursors and their requirement of sphingosine-1-phosphate receptor 1 for heart development
Source: Sci Rep. 2017 Mar 24;7:45205. doi: 10.1038/srep45205 (PMC5364549; doi:10.1038/srep45205)
Supplement: Supplementary Information [file srep45205-s2.pdf]

Identification of cardiac hemo-vascular precursors and their requirement of sphingosine-1-phosphate receptor 1 for heart development

Yan Hu [1], Brian C. Belyea[1], Minghong Li[1], Joachim R. Göthert [2], R. Ariel Gomez[1] and Maria Luisa S. Sequeira-Lopez[1*]

[1]Department of Pediatrics and Department of Biology, University of Virginia, Charlottesville, Virginia

[2]Department of Hematology, West German Cancer Center, University Hospital Essen, Essen, Germany.

Correspondence :

Maria Luisa S. Sequeira Lopez, M.D.

Professor of Pediatrics
University of Virginia School of Medicine
409 Lane Rd MR4 Bldg room 2010
Charlottesville, VA 22908
msl7u@virginia.edu
Phone: 434-924-5065
Fax: 434-924-8936

Running title: Hemo-vascular precursors in the developing heart

Supplemental Figures And Figure Legends

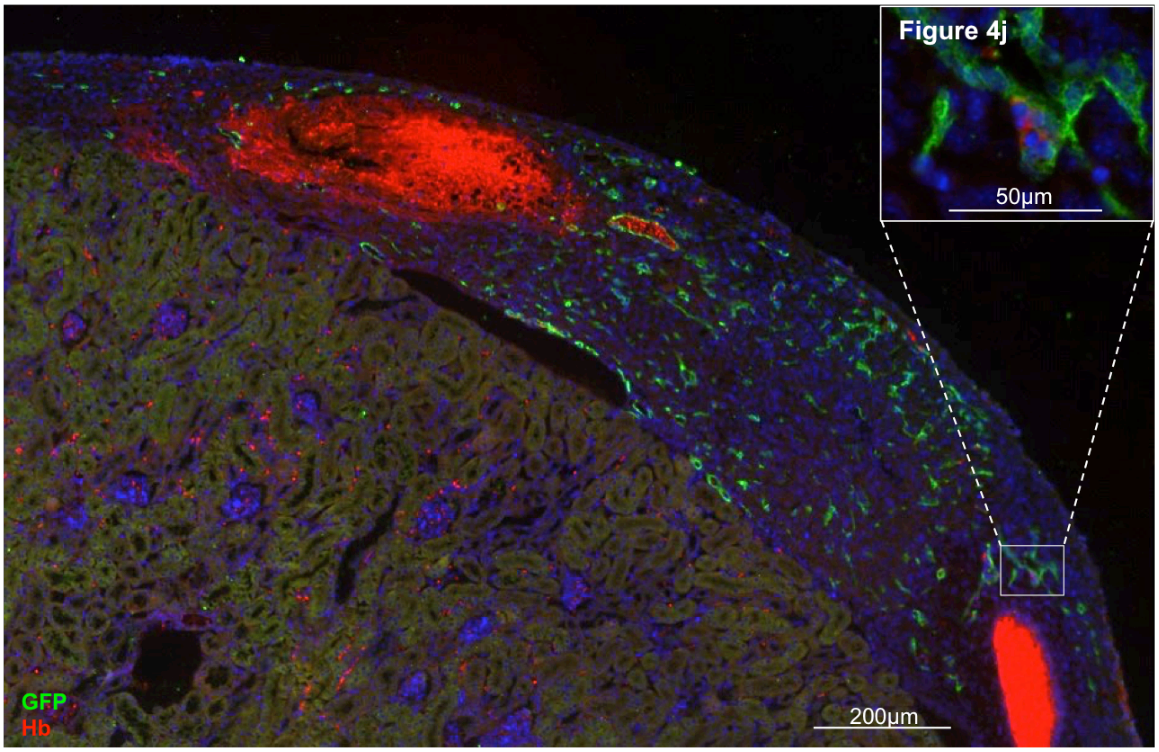

Supplemental Figure 1 related to Figure 4. Ablation of SCL^{EC}+ precursors impairs hemo-vasculogenesis and heart development. Low magnification image of a section stained by IF for GFP and Hb of a control transplanted heart showing the location of Figure 4j. (white box)

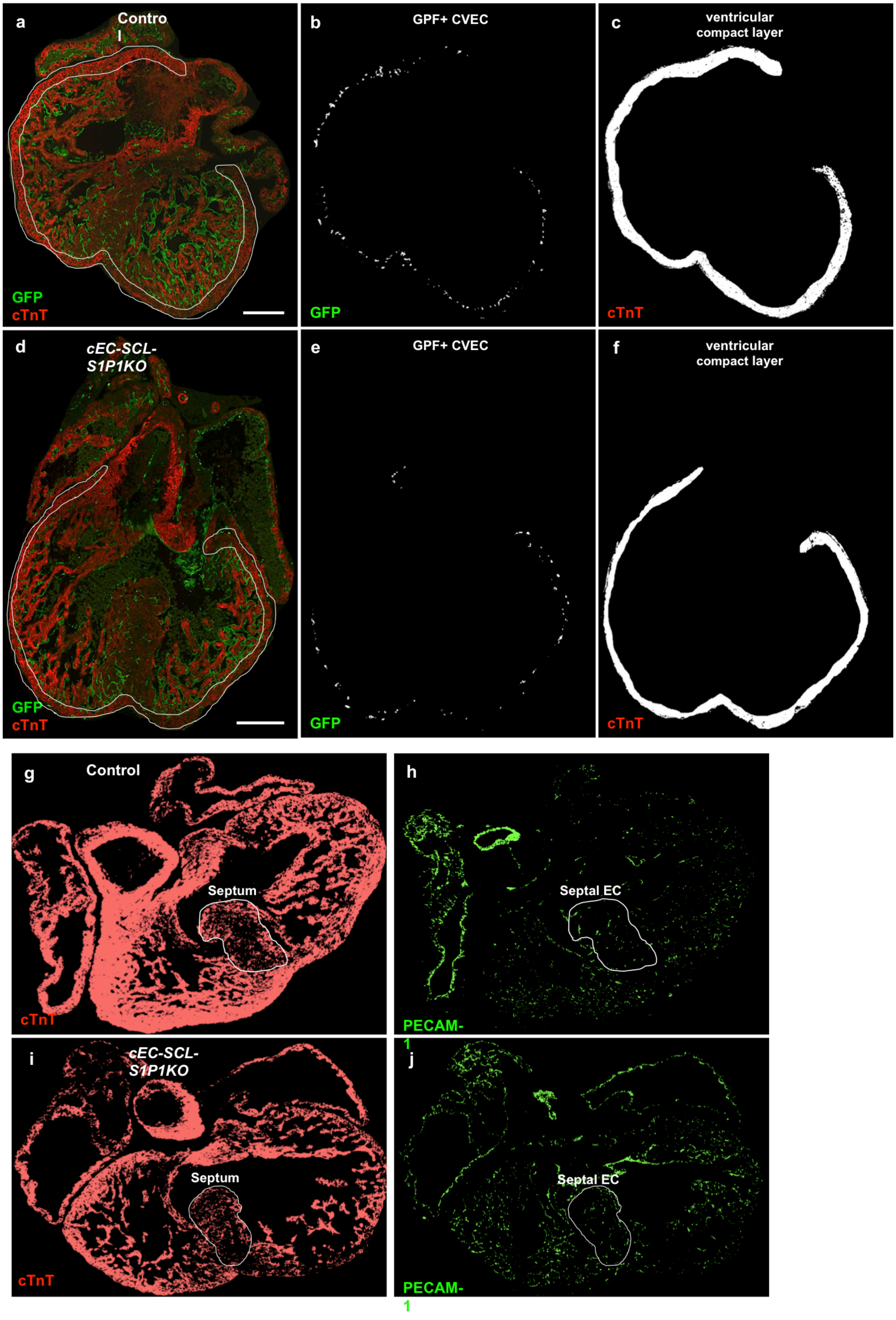
a
Contro
l
GFP
cTnT
b
GPF+ CVEC
GFP
c
ventricular
compact layer
cTnT
d
cEC-SCL-
S1P1KO
GFP
cTnT
e
GPF+ CVEC
GFP
f
ventricular
compact layer
cTnT
g
Control
Septum
cTnT
h
Septal EC
PECAM-
1
i
cEC-SCL-
S1P1KO
Septum
cTnT
j
Septal EC
PECAM-
1

Supplemental Figure 2 related to Figure 5. *S1P1* in *SCL*^{EC}+ precursors is crucial for normal heart development.

[a-f] Measurement of GFP+ CVEC area in the ventricular compact layer in heart sections of E12.5 *control* (a-c) and *cEC-SCL-S1P1KO* (d-f) mice. The measured ventricular areas are shown in the white lined area of a and d. b and e show GFP+ CVECs in the defined areas. c and f show defined cTnT+ ventricular compact layer. The areas of GFP+ cells and cTnT cells in the ventricle were measured using image J.

[g-j] Measurement of PECAM-1+ cells/cTnT+ cells was performed in the septum areas defined by the white lined area of heart sections from E12.5 *control* (g and h) and *cEC-SCL-S1P1KO* (I and j) mice. The areas of PECAM-1+ cells (h and j) and cTnT+ cells (g and i) within the septum area were measured using imageJ.

Scale bars: 200μm

Video Legend

Movie 1. Beating transplanted heart under host kidney capsule. Video shows beating embryonic heart (arrow) under host kidney capsule at day 7 post transplantation.
